# Deciphering Diseases and Biological Targets for Environmental Chemicals using Toxicogenomics Networks

**DOI:** 10.1371/journal.pcbi.1000788

**Published:** 2010-05-20

**Authors:** Karine Audouze, Agnieszka Sierakowska Juncker, Francisco J. S. S. A. Roque, Konrad Krysiak-Baltyn, Nils Weinhold, Olivier Taboureau, Thomas Skøt Jensen, Søren Brunak

**Affiliations:** Center for Biological Sequence Analysis, Department of Systems Biology, Technical University of Denmark, Lyngby, Denmark; University of Notre Dame, United States of America

## Abstract

Exposure to environmental chemicals and drugs may have a negative effect on human health. A better understanding of the molecular mechanism of such compounds is needed to determine the risk. We present a high confidence human protein-protein association network built upon the integration of chemical toxicology and systems biology. This computational systems chemical biology model reveals uncharacterized connections between compounds and diseases, thus predicting which compounds may be risk factors for human health. Additionally, the network can be used to identify unexpected potential associations between chemicals and proteins. Examples are shown for chemicals associated with breast cancer, lung cancer and necrosis, and potential protein targets for di-ethylhexyl-phthalate, 2,3,7,8-tetrachlorodibenzo-p-dioxin, pirinixic acid and permethrine. The chemical-protein associations are supported through recent published studies, which illustrate the power of our approach that integrates toxicogenomics data with other data types.

## Author Summary

Exposure to environmental chemicals and drugs may have a negative effect on human health. An essential step towards understanding the effect of chemicals on human health is to identify all possible molecular targets of a given chemical. Recently, various network-oriented chemical pharmacology approaches have been published. However, these methods limit the protein prediction to already known molecular drug targets. New findings can for example be made by using high-confidence protein-protein association databases. Here, we describe a generic, computational systems biology model with the aim of understanding the underlying molecular mechanisms of chemicals and the biological pathways they perturb. We present a novel and complementary approach to existing models by integrating toxicogenomics data, chemical structures, protein-protein interaction data, disease information and functional annotation of proteins. The high confidence protein-protein association network proposed reveals unexpected connections between chemicals and diseases or human proteins. We provide literature support to demonstrate the validity of some predictions, and thereby illustrate the power of an approach that integrates toxicogenomics data with other data types.

## Introduction

Humans are daily exposed to diverse hazardous chemicals via skincare products, plastic cups, computers and pesticides to mention but a few sources. The potential effect of these environmental compounds on human health is a major concern [1]–[2]. For example chemicals such as phthalate plasticizers have been widely linked to allergies, reproductive disorders and neurological defects. Humans are intentionally exposed to drugs used for treatment and cure of diseases. Many drugs affect multiple targets and may interact or affect the same proteins as environmental chemicals [3]–[5]. The mechanism of action of these small molecules is often not completely understood and can be associated to adverse and toxic effects through for example drug-drug interactions [6]. There is thus a need to improve our understanding of the underlying mechanism of action of chemicals and the biological pathways they perturb to fully evaluate the impact of small molecules on human health.

An essential step towards deciphering the effect of chemicals on human health is to identify all possible molecular targets of a given chemical. Various network-oriented chemical pharmacology approaches have been published recently to identify novel protein candidates for drugs, using structural chemical similarity [7]–[10]. For example Keiser *et al.* [8] applied network analysis to drugs and their targets. The authors identified unexpected molecular targets such as muscarinic acetylcholine receptor M_3_, alpha-2 adrenergic receptor and neurokinin NK2 receptor for methadone, emetine and loperamide, respectively. Additionally, recent studies have demonstrated that chemicals could be classified based upon their effect on mRNA expression detected by microarrays [11]–[12]. Lamb et al. showed that genomic signatures could be used to recognize drugs with common mechanism of action allowing discovery of unknown modes of action. Despite the explosion of chemical-biological networks, the chemical toxicity remains a major issue in human health. Analysis of environmental chemicals with similar gene expression profiles is still lacking. With the recent advances in toxicogenomics, information on gene/protein activity in response to small molecule exposures becomes more available. This provide necessary data to develop computational systems biology models to predict both high level associations (linking chemical exposures to diseases) and more detailed associations (linking chemicals to proteins)

In this paper we present a method that can associate chemicals to disease and identify potential molecular targets based on the integration of toxicogenomics data, chemical structures, protein-protein interaction data, disease information and functional annotation. The core of our procedure is derived from the “target hopping” concept defined previously [3]. But instead of considering only binding activity, we extended the concept to gene expression. If two proteins are affected with two chemicals, then both proteins are deemed associating in chemical space. Our approach is not only a statistical model but mimics the true biological system by constructing a network of associations between human proteins defined as Protein-Protein Association Network (P-PAN). We have validated our network by comparison with two high confidence protein-protein interaction (PPI) networks, and by assessing the functional enrichment of clusters in the network generated. The P-PAN revealed both known as well as many novel surprising connections between chemicals and diseases or proteins. We provide literature support for some of the unexpected associations, such as the connection between diethylhexylphthalate (DEHP) and gamma-aminobutyric acid A receptor beta target [13], as well as between apocarotenal, a chemical found in spinach, and necrosis. This illustrates the usefulness of an approach that integrates toxicogenomics data with other diverse data types.

## Results

**Figure 1:**
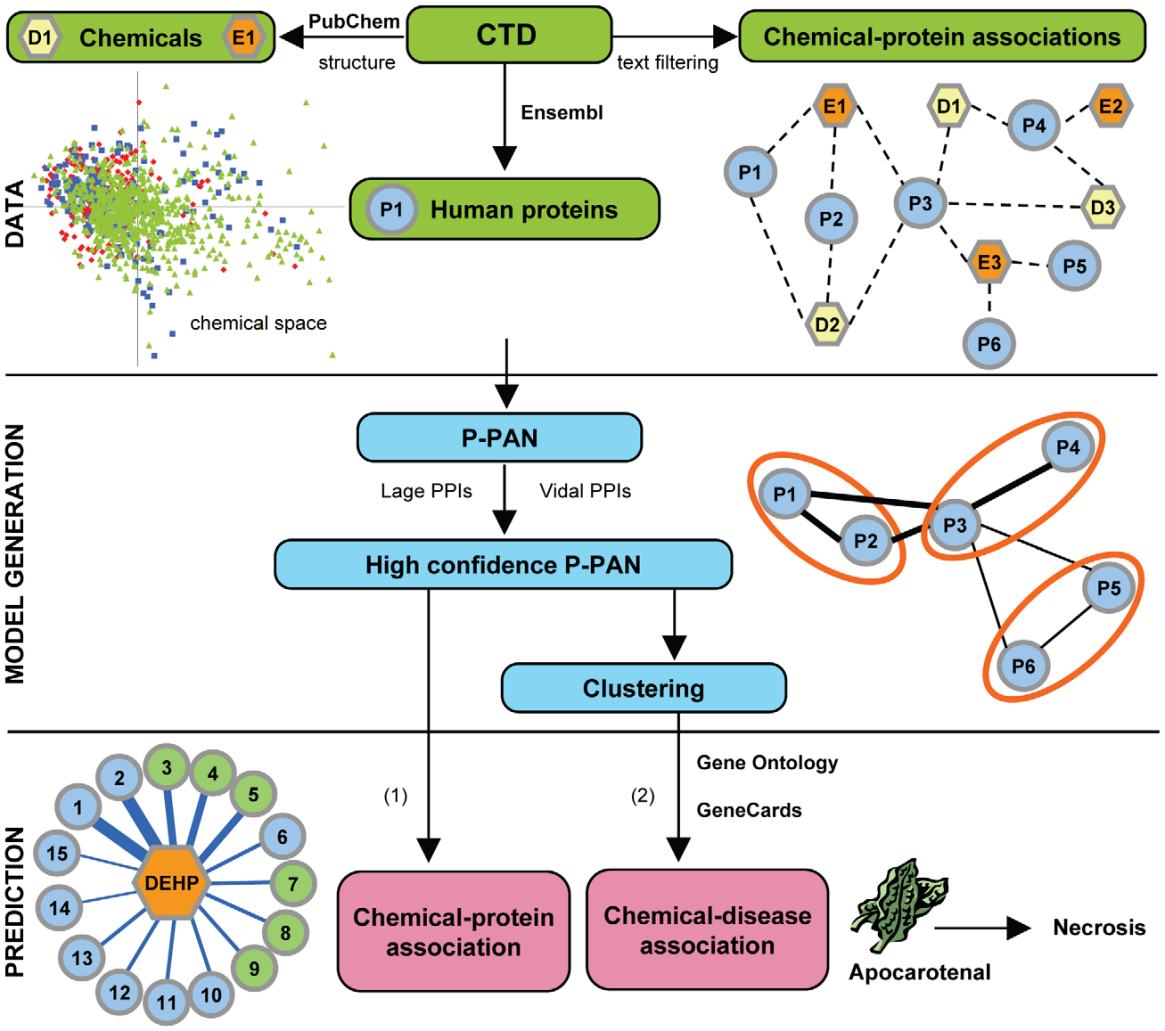
Workflow of the strategy for generating a human P-PAN and predicting novel associations. DATA: Extraction and filtering of human protein-chemical associations from CTD. The visualization of the chemical space by Principal Component Analysis projection confirms that drugs (D) and environmental chemicals (E) shared structural properties, and then may affect similar protein targets. The two first principal components, which explained about 44% of the variance on the calculated properties are shown (green: pharmaceutical actions, red: toxic actions and blue: specialty uses of chemical). All proteins (P) were mapped to Ensembl gene identifiers to facilitate further data integration. MODEL GENERATION: Construction of the P-PAN. The P-PAN was created from associations present in the CTD (dashed edge lines) between chemicals and proteins. In the P-PAN, two proteins are connected to each other (edge lines) if they share a common chemical. A weighted score, represented by the width of the black edges, was assigned to each protein-protein association. It represents the strength of the network between two proteins as defined by the number of shared compounds for both molecular targets. Selection of a scoring function and a high confidence P-PAN after overlaps comparison with two human interactomes (PPIs) based on experimental evidences. Clustering of the P-PAN and evaluation of the biological meaningful of the clusters using Gene Ontology annotations. PREDICTION: (1) Prediction of novel molecular targets for chemical using a neighbor protein procedure. DEHP (orange) is known to be connected with blue proteins and is predicted to be associated with green proteins. A confidence score was calculated for each protein, represented by the width of the edges; thick edge for high score to thin edge for low score. (2) Prediction of disease associated with chemical after integration of protein-disease information using GeneCards in clusters. As example, apocarotenal, a compound found in spinach is predicted to be link to necrosis.

Based on the Comparative Toxicogenomics Database (CTD) [14], we constructed a human P-PAN. A workflow of the strategy is shown on Figure 1. We extracted 42,194 associations between 2,490 chemicals and 6,060 human proteins from the CTD. We mapped compounds to chemical structures from PubChem and extracted their indication of use from Medical Subject Headings (MeSH, http://www.nlm.nih.gov/mesh/MBrowser.html) to classify them as either drugs (MeSH: “Pharmaceutical Actions”) or environmental chemicals (MeSH: “Toxic Actions” and “Specialty Uses of Chemicals”).

In the CTD, drugs and environmental compounds are claimed to be associated with toxicologically important proteins. To estimate how much the information from the CTD differs from available data on pharmacological action of drugs, we compared the data shared between CTD and DrugBank, as of May 2009 [15]. DrugBank is a repository of pharmacological action for ‘Food and Drug Administration’ approved drugs. From the 1358 drugs gathered in DrugBank, 420 drugs matched in CTD. Interestingly, whereas 1403 proteins are associated to these drugs in DrugBank, only 194 proteins are found in both databases. For example, according to Drug Bank celecoxib, a known non-steroidal anti-inflammatory drug, is associated to two metabolizing enzymes: the Cytochrome P450 2C9 (CYP2C9) and the Cytochrome P450 2D6 (CYP2D6) and to two drug targets: the Prostaglandin G/H synthase 2 (COX-2) and the 3-phosphoinositide-dependent protein kinase 1 (PDPK1). In the CTD, celecoxib is linked to 33 human proteins including CYP2C9 and COX-2. The toxicity information extracted from CTD is relatively different to the known pharmacological action of drugs and should be considered as a complementary source of information.

### Structure-target relationship

To investigate the assumption that two compounds sharing similar structure can potentially affect the same molecular targets, we compared chemical properties of the compounds collected from the CTD. The chemicals were characterized by 50 properties calculated from the structure, including the molecular mass and affinity for a lipid environment. The distribution of properties, as it appears in a multi-dimensional properties space, was projected and visualized in two dimensions using principal component analysis (PCA) (shown in Figure 1). There is substantial overlap in the PCA projections between environmental chemicals and drugs indicating that they can potentially affect the same protein targets. We also compared the oral bioavailability profiles of compounds based on standard Lipinski [16] and Veber [17] rules. Again, overlaps were observed, indicating that environmental chemicals mimic drug properties (see Figure S1). These results confirm that it is reasonable to generate a network by integrating toxicogenomics knowledge from both drugs and environmental compounds, as they share many properties.

### Generating a high confidence human Protein-Protein Association Network

The human P-PAN was generated based on the assumption that if two proteins are biologically affected with the same chemicals (defined as shared chemicals), they are likely to be involved in a common mechanism of action of the chemicals. Then, two proteins are connected to each other if they are linked to the same chemical in the CTD. The resulting P-PAN consists of 2.44 million associations. To reduce noise and select the most significant associations, we assigned two reliability scores to each protein-protein association: a score based on hypergeometric calculation and a weighted score. The weighted score was calculated as the sum of weights for shared chemicals, where weights were inversely proportional to the number of associated proteins for a given compound.

We went one-step further and compared the P-PAN with two human PPI databases: (1) a high confidence set of experimental PPIs extracted from a compilation of diverse data sources [18] and (2) PPIs based on an internal consistent single data source [19]. Our P-PAN performed well compared to both PPIs. Based on the calibration curves (Figure S2), we considered a threshold that capture good overlaps between our P-PAN and the PPI networks for different reliability scores thus reducing our P-PAN to ∼200,000 reliable associations. Using this approach, the molecular target predictions are limited to the 3,528 proteins present in the P-PAN. To confirm that biological information is not lost when selecting only 8% of the entire P-PAN, we compared functional enrichment for the complete network (6,060 proteins) and for the high confidence sub-network (3,528 proteins) using Gene Ontology (GO) [20]. For example cell proliferation (p-values of 3.22e-36 and 1.46e-27 for the large network and the sub-network, respectively) and protein binding (p-values of 1.2e-72 and 4.13e-47 for the large network and the sub-network, respectively) were the most overrepresented terms.

Since proteins tend to function in groups, or complexes, an important step has been to verify that our high confidence network mimics true biological organization. This task is commonly executed using graph clustering procedures, which aim at detecting densely connected regions within the interaction graph. Two clustering methods have been applied to our network. The molecular complex detection (MCODE) approach [21] that allows multiple clusters assignation for a protein, mimicking the reality as a protein can participate in several complexes simultaneously. On the other hand, the markov cluster algorithm (MCL) [22] which assign one protein to a unique cluster has been shown to be superior to other graph clustering methods in recent studies [23]–[24]. Applied on our network, MCODE extracted few large core clusters and several tiny clusters (possibly singleton clusters). The MCODE approach results in a clustering arrangement with a weak cluster-wise separation. Compared to MCL, MCODE yielded a lower number of clusters, with a higher number of proteins per cluster. Only 35 clusters varying in size from five to 845 proteins were extracted. Using the MCL algorithm we obtained a more heterogeneous separation with 58 clusters varying in size from five to 462 proteins. Therefore, to identify the biologically meaningfulness of our network, we used complexes extracted using the MCL method. Each cluster was then investigated for functional enrichment based on GO terms. To ensure the high quality of functional annotations we used only annotations experimentally supported or with traceable references. Hypergeometric testing was used to determine GO functional annotation overrepresented amongst each cluster. The two top scoring molecular functions found were heme binding (p-value of 6.60e-25, cluster 4) and glucuronosyl transferase activity (p-value of 2.34e-21, cluster 12). Regulation of apoptosis (p-value of 1.67e.17, cluster 2) and oxidation reduction (p-value of 6.67e-14, cluster 4) were the most highly enriched categories in the biological process branch of the GO. This analysis thus confirms that clusters in the network, and therefore the proteins associated with each other, are functionally coherent. This was further evidence that the organization of the network is meaningful.

### Diseases associated to clusters

In the clusters of the P-PAN, proteins are more connected with other proteins within the cluster than with the other targets in the network. As proteins are associated based on their shared relationship with chemicals, proteins within a given cluster tend to be more linked to specific compounds. It is thus possible to find associations between diseases and the chemicals that underlie the protein-protein associations within the cluster using protein-specific disease annotations. For each cluster, we investigated if specific disease annotation was found more frequently than expected by using protein-disease information [25]. We identified several diseases associated with specific clusters. These included the two most common types of cancer, breast cancer (cluster 1, p-value of 9.67e-18) and lung cancer (cluster 12, p-value of 4.84e-12), as well as necrosis (cluster 2, p-value of 2.26e-12), ichthyosis (a skin disorder associated to cluster 4, p-value of 1.41e-5), retinoblastoma (cluster 7, p-value of 9.46e-8) and inflammation (cluster 8, p-value of 1.55e-5).

### Mining the network for chemicals associated with disease

To predict which chemicals may affect human health, we then analyzed selected clusters to identify new chemical-disease associations (see Table 1). When linking diseases to compounds, it is important to keep in mind that there is no direction in the association, i.e. it is not possible from the network to separate positive from negative associations between a chemical and a disease. Discriminating between whether a compound prevents or causes disease requires manual interpretation of the association.

**Table 1:** Mining the P-PAN for chemicals associated with breast cancer, lung cancer and necrosis, using a clustering procedure.

Cluster ID	Disease	Chemical name	p-Value
1 (462 proteins)	Breast cancer (128 proteins)	*estradiol*	7.68e-134
		*bisphenol A*	4.46e-92
		*PCBs*	1,15e-88
		*genistein*	2.20e-78
		*fulvestrant*	7.05e-63
12 (59 proteins)	Lung cancer (29 proteins)	**thimerosal**	1.57e-26
		(10 proteins)	
		**DNCB**	3.29e-22
		(12 proteins)	
		*styrene*	7.78e-06
2 (433 proteins)	Necrosis (122 proteins)	*arsenic disulfide*	4.76e-35
		**apocarotenal**	1.63e-29
		(8 proteins)	
		*doxorubicin*	2.66e-26

Chemicals already known from the literature to be associated to disease are shown in italic. In bold are the chemicals significantly associated to disease, which are unknown to be disease-causing chemical from the literature. The number of proteins is shown in brackets for each cluster, disease and novel association. As example, among the 433 proteins associated to cluster 2, 122 are known to be linked to necrosis. Among these 122, 8 are connected to apocarotenal in CTD.

One of the clusters showed high enrichment for breast cancer. The most significantly associated chemicals are already known from the literature to be related to cancer, thus supporting the clustering quality of the P-PAN. Among the most significantly associated chemicals are the well-known polychlorinated biphenyls (PCBs). PCBs are used for a variety of applications i.e. flame retardants, paints and plasticizers. After being banned due to their toxicity, they still persist in the environment. Previous results suggest that specific PCBs may indeed be associated with breast cancer [26]. Several organizations (EPA, IARC) have classified PCBs as probable human carcinogens. When we inspected another cluster highly connected to lung cancer using our P-PAN method, thimerosal, dinitrochlorobenzene (DNCB) and styrene were significantly associated with this cluster. Thimerosal and DNCB are not known lung cancer-causing chemicals, while the last compound, styrene has been classified as a possible carcinogen. Thimerosal is an organomercury chemical widely used as preservative in health care products and in vaccines. It may have possible adverse health effects such as a role in autism and in nervous system disorders [27] as well as possible gene-toxic effects to human lymphocytes [28]. No study has previously related it to lung cancer. The second chemical DNCB is known to be a skin allergen that may cause dermatitis. Genes associated with allergies were shown to be up regulated in rat lung tissue after DNCB exposure [29], but no direct link to lung cancer has been demonstrated so far. Another interesting finding is the association between apocarotenal and necrosis. Apocarotenal, a natural carotenoid found in spinach and citrus, is used as a red-orange coloring agent (E160E) in foods, pharmaceuticals and cosmetics products. No direct evidence has been found that links apocarotenal to necrosis. However, *in vitro* and *in vivo* studies [30] have suggested that spinach may be a good anti-cancer agent. This is in line with epidemiologic studies that have shown that those who consume higher dietary levels of fruits and vegetables have a lower risk of certain types of cancer [31] due to the presence of carotenoids. Furthermore, carotenoids have been defined as chemopreventive agents [32]. Studies have established associations between carotene and beta-carotene with reduced risk of prostate cancer [33] or breast cancer [34]. The prediction that apocarotenal is positively associated to necrosis and could prevent certain types of cancer is thus indirectly supported by other studies. The other chemicals significantly associated to disease (Table 1) are discussed in the supplementary text (see Text S1).

### Predicting novel molecular targets for chemicals

Besides revealing disease-chemical associations, the network can be used to predict novel targets for chemicals. It has been shown that many small molecules affect multiple proteins rather than a single target, and that proteins sharing an interaction with a chemical are targeted by the same chemicals [8]. Based on the CTD data available, strong promiscuities between some proteins exist. For example, more of 25% of chemicals annotated to estrogen receptor 1 (ESR1) affects also progesterone receptor (PGR). In the same order, cytochrome p450 2D6 (CYP2D6) and cytochrome p450 2C9 (CYP2C9) shared one-third of their respective associated compounds. By the term “affected”, we consider effects such as up regulated, down regulated, agonist, antagonist and inhibitor. Then, our network can not be used to identify chemical synergies or opposite effect on proteins. Thus, if two proteins are affected by two chemicals and one of the proteins is further deregulated by an additional chemical, then it might be that both proteins are in fact deregulated with the same three chemicals. Based on this assumption and in order to suggest novel associations between chemicals and proteins, a neighbor protein procedure was used which scored the association between each protein and each chemical (see Materials and Methods). Molecular targets known to be associated with a chemical were extracted from the CTD, and the P-PAN was scanned for proteins associated with a high score. The significance of enrichment was calculated by random testing (for the confidence scores see Text S2), and sub-networks were subsequently ordered according to their significance. Four examples of various chemicals are presented in Table 2 (other case stories are shown in Table S1). To estimate the performance of our approach for approved drugs, we analyzed the level of recall and precision obtained for the 420 common drugs between DrugBank and CTD. We obtained a recall and a precision of 5.91% and 3.77% respectively, corresponding to the percentage of interactions in DrugBank retrieved and percentage of interactions in DrugBank from all interactions predicted obtained from CTD data and from the neighbor protein procedure. These values illustrate that information between the two data sources are relatively different.

**Table 2:** Predicting novel molecular targets for chemicals.

Chemical	Known protein	Cpscore*	Novel protein	Cpscore*	Literature
DEHP	CDO1	13.23	**GABAß1**	5.46	Yes
	PPARA	9.48	**POMC**	5.44	Yes
	SUOX	4.35	**CYP3A11**	5.40	Yes
	(15 proteins)		**GABAß2**	4.32	Yes
			**GABAγ2**	4.32	Yes
			**GABAα1**	4.26	Yes
TCDD	HSPA9B	82.69	**PRKCE**	10.17	Yes
	SLC2A4	82.69	**POMC**	8.97	Yes
	TRIP11	82.69	**CPT1A**	6.96	Yes
	TSP1	82.69	**HSD11B1**	6.39	Yes
	EPHX2	75.77	***MVP***	6.77	No
	MT2A	10.85	**APOB**	5.61	Yes
	(90 proteins)				
PA	CYP4X1	5.67	***CHST1***	5.19	No
	PPARA	2.53	***CHST4***	5.19	No
	CES1	1.45	**CST**	3.19	Yes
	SULT2A1	0.87	***ABCG5***	2.61	No
	CYP1A1	0.37	**C3**	2.80	Yes
			**ADRA2A**	1.34	Yes
			***CYB5A***	1.21	No
			**ADRA1A**	1.08	Yes
			***CRHR2***	1.04	No
			***CYP2A13***	0.93	No
			**ALDH3**	0.91	Yes
	(5 proteins)				
Permethrin	AR	4.67	**CYP2B1**	4.43	Yes
	WNT10B	4.12	**SHBG**	3.51	Yes
	PGR	3.75	***CYP2B6***	2.89	No
	ESR1	3.31	**NR1I3**	2.64	Yes
	TFF1	3.15			
	NR1I2	2.94			
	(17 proteins)				

*Proteins known to be associated to a compound were extracted from the CTD. In brackets is the total number of known proteins used to query the P-PAN. To find novel protein targets (in bold) associated to a chemical, a neighbor proteins procedure was used which scored the association between proteins and chemicals (cpscore). Among the novel predicted proteins (thus not input data), some are supported by literature, highlighting the usefulness of the P-PAN to identify new chemical-protein associations.

### Examples of proteins associated to chemicals

Phthalates, mainly used as plasticizers, have received a lot of attention as environmental compounds because they are potential human carcinogens. As there are many phthalates, we focused on Di-EthylHexyl Phthalate (DEHP) that has been associated with more proteins compared to other phthalates such as additional information on kinases (e.g. mitogen-activated protein kinase 1, and mitogen-activated protein kinase 3) [35]. DEHP is widely used due to its suitable properties and low cost, and is present in the general environment at high levels. Exposure to DEHP is of particular concern with regard to developing fetuses where it is believed to cause malformation of reproductive organs and neurological defects [36]. Using our approach, several proteins were identified as being associated with DEHP (Table 2). Cysteine dioxygenase type I (CD01) and peroxisome proliferator-activated receptor alpha (PPARA), the two top scoring proteins, are already known in the CTD and from the literature [37]–[38] as molecular targets for DEHP. Six other high ranking proteins are new potential DEHP molecular targets which are not recorded in the CTD (thus not input data). Among them, four gamma-aminobutyric acid A (GABA) receptors were predicted as potential DEHP molecular targets. These associations are supported by a recent study showing that DEHP can modulate the function of ion channels as GABA receptors in a manner similar to volatile anesthetics in experiments on expressed receptors [13]. This makes sense because the GABA neurotransmitter system has been implicated in the pathogenesis of bipolar disorders (neurological disorders) via gamma-aminobutyric acid receptor subunit alpha-1 (GABAα1) [39], and DEHP is also associated with neurological defects [36]. In addition to GABA receptors, we identified several other candidates including proopiomelanocortin (POMC) and a cytochrome P450 (CYP3A11) (discussed in the Text S2). We looked at another environmental chemical, the 2,3,7,8-TetraChloroDibenzo-p-Dioxin (TCDD), which originates from burning or incineration of chlorinated industrial compounds. TCDD is believed to cause a wide variety of pathological alterations, with the most severe being progressive anorexia and body weight loss [40]. TCDD is also known to be a neurotoxin leading to neurodevelopmental and neurobehavioral deficits [41]–[42], and accumulating in the brain as well as other organs [43]. We identified six proteins associated with TCDD that are not recorded in the CTD for human (Table 2). Among them five are supported by literature (see Text S2). This included protein kinase C elipson (PRKCE), known to be involved in brain tumors [44], carnitine palmitoyltransferase I (CPT1A), 11β-hydroxysteroid dehydrogenase type 1 (HSD11B1) and apolipoprotein B (APOB) which are all linked to obesity [45]–[47]. Furthermore, we investigated in detail the drug pirinixic acid (PA) (also named WY14,643), which is a peroxisome proliferator-activated receptor (PPAR) agonist with strong hypolipidemic effects. PA was never approved for clinical use due to hepatocarcinogenesis adverse effect shown in animal studies [48]. To date there is no evidence that PA promotes carcinogenesis in humans [49], and this has spurred new studies for identifying cellular processes that are capable of responding to PA. Among 11 molecular targets identified and not recorded in the CTD (Table 2), only five are supported by the literature (see Text S2). For example the expression of the C3 protein, an acylation stimulating protein involved in necrosis and afibrinogenemia (blood disorders), has been shown to be affected by PA in rats [50]. Finally we studied proteins associated with permethrin in more detail. Permethrin is a widely used insecticide, acaricide and insect repellent, classified by the US EPA as a likely human carcinogen, but still used in healthcare for the treatment of lice infestations and scabies. Four proteins not recorded in the CTD were identified as associated with permethrin. Three of them are supported by literature (see Text S2 for details) including a cytochrome P450 (CYP2B1) [51]–[52] and sex hormone-binding globulin (SHBG) [53], which are proteins linked to the endocrine system. These findings suggest a mechanism by which chronic exposure of humans to pesticides containing this compound may result in disturbances in endocrine effects related to androgen action.

The examples we provide include both known and new protein associations with a given chemical, and many of the novel associations are supported by the literature. We compared our approach with STRING (version STRING 1) [54] a high-confidence protein-protein association network, to see if the findings generated by the current approach are also found by other existing methods. The STRING network includes direct (physical) and indirect (functional) associations derived from diverse sources as genomic context, high throughput experiments, co-expression and literature. As a test example, we used the 15 proteins associated with DEHP in the CTD to query the P-PAN by a neighbor protein procedure. The same 15 proteins were also used to query the STRING network. Subsequently we compared the predicted molecular targets between the two networks (P-PAN and STRING). In the resulting STRING network none of the GABA receptors were found (see Figure S3). The STRING network showed a clear tendency to associate phthalates with kinases and nuclear receptors. This example demonstrated that our approach was complementary to other association approaches. This highlights the value of integrating various sources of data to understand potential toxic effects on human health caused by chemical exposure.

## Discussion

We propose an approach different from existing computational chemical biology networks, which primarily integrate drugs information, to identify new molecular targets for chemicals and to link them to diseases. In our approach we have integrated toxicogenomics data for drugs and environmental compounds. The ability to make new findings using a different network is illustrated by a comparison with a similar method, showing the capacity of our P-PAN to identify novel chemical-protein associations. Using phthalate as an example, our model suggests potential associations between DEHP and GABA receptors, which have not been predicted previously.

An extension of this network by integrating more data, for example other chemical-protein associations or dose levels for which a compound may affect human health, would be beneficial to the proposed approach. Paracelsus (1493–1541) is often cited for his quote, “all things are poisons and nothing is without poison, only the dose permits something not to be poisonous”. This emphasize that the dose of a chemical is an issue to consider in the deregulation of systems biology. Nevertheless, a global mapping could allow a better understanding of adverse effects of drugs and toxic effects of environmental compounds. This could be used as a new approach for risk assessment and regulatory decision-making for human health.

**Figure 2:**
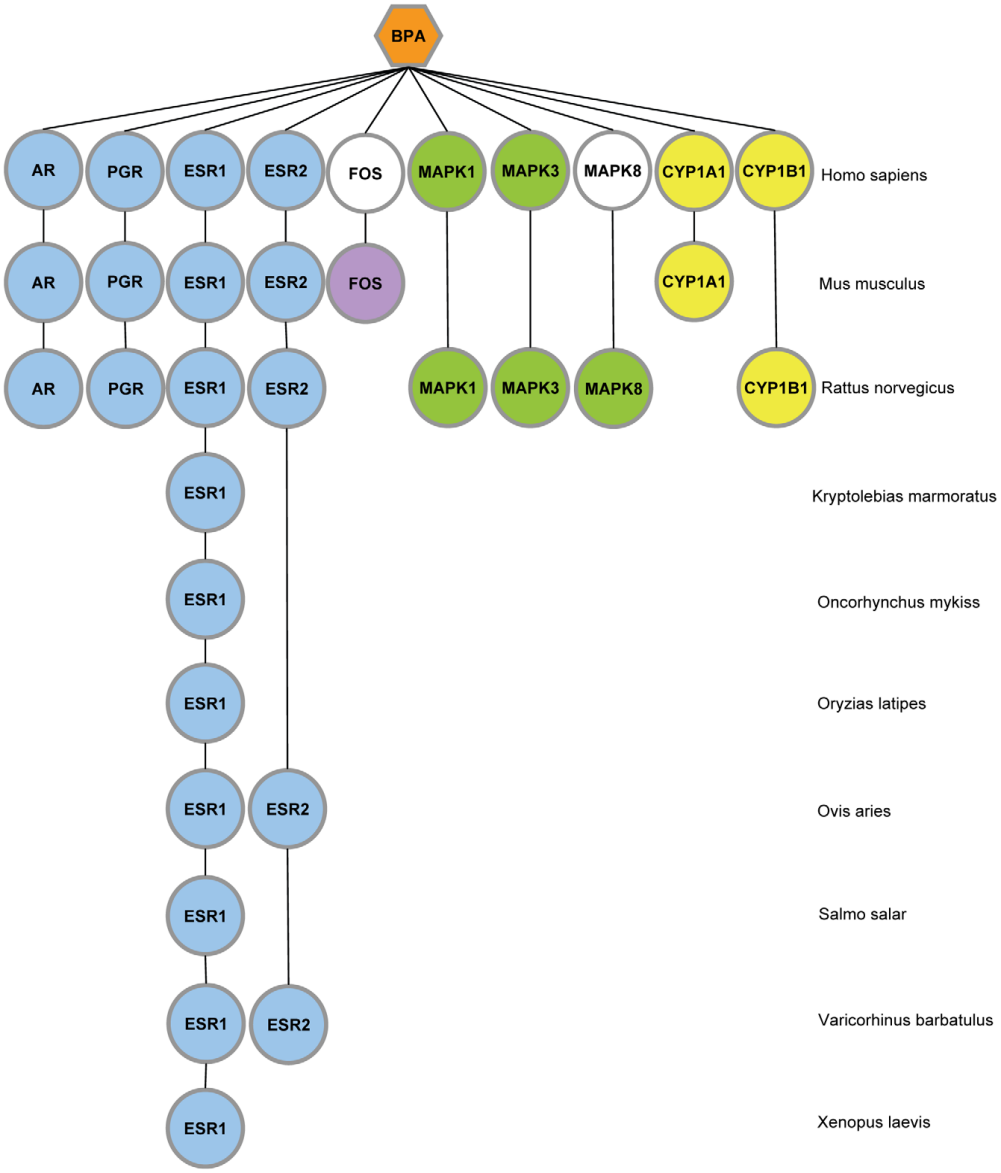
Cross-species comparative toxicogenomics for bisphenol A (BPA). Molecular targets are represented as nodes, and colored by gene family. Nodes presence represent available information extracted from the CTD and node absence are the unknown information. Colored nodes defined that BPA affect the protein, while nodes are not colored when BPA does not affect the protein. This figure highlights similarities and differences existing between animal model and human responses to chemical exposure.

Among the examples presented, some predictions are supported by literature for other organisms. Regarding toxicogenomics, the available human data are generally sparse compared to rodents. Data on toxicity - adverse effects of chemicals on humans – can be acquired through epidemiologic studies and from occupational, accident-related exposures as intentional human testing of environmental compounds remains limited. However, differences exist between model animal and human responses to chemicals, including differences in the type of adverse effects experienced and the dosages at which they occur. The differences may reflect variations in the underlying biochemical mechanisms, in metabolism, or in the distribution of the chemicals. As an example, bisphenol A (BPA) does not affect proteins in a similar way across species (Figure 2). In the human systems studied to date, BPA does not affect the proto-oncogene c-FOS (FOS) and the mitogen-activated protein kinase 8 (MAKP8) but seems to modify their expression in rodent species. BPA binds and modifies the activity of the estrogen receptor alpha (ESR1) in a very conservative way across organisms [14]. BPA has an ability to function as an estrogen like receptor (ER) agonist, and thus has the potential to disrupt normal endocrine signaling through regulation of ER target genes e.g. androgen receptors, estrogen receptor, progesterone receptors. There is a need to integrate data with cross-species extrapolation in order to have a more accurate understanding of the human risk from chemical exposure.

The major limitation of our integrative systems biology approach is that the molecular target predictions are limited to the 3,528 proteins present in our P-PAN, which represent only 15% of the estimated human proteome [55]. Hence, the current lack of high quality data is the limiting factor in approaches such as the one described here. Today high throughput methodologies result in available large scale data in both chemical biology and systems biology, but these data are discipline specific [56]. There is an evident need for the development of databases [57] to integrate disparate datasets such as toxicogenomics data in order progress in systems biology research. In addition, the results of the disease-compound association analysis will improve in the future as newer, more complete and curated data will become available.

## Materials and Methods

### Data set

We downloaded the publicly available Comparative Toxicogenomics Database (CTD) as of June 26, 2008 [14]. The CTD contains curated information combining drug and environmental chemical data associated with proteins. We selected 42,194 associations between 2,490 unique compounds and 6,060 molecular targets known to be involved in human disease. Different associations are presented in the CTD such as “chemical x results in increased expression of protein z” or “compound x binds to protein z”. Gene expression data are essentially present in the CTD such as a chemical can increase, decrease or affect a gene expression. However, only few binding data are present in CTD and therefore integrated in our network: 3189 in total among the 42,194 associations. Scripts were used to remove associations with negation such as “chemical x does not affect protein z”.

### Quality of chemical and protein annotations

To verify the uniqueness of chemicals, chemical names extracted from the CTD were checked using PubChem (http://pubchem.ncbi.nlm.nih.gov/) as of June 26, 2008 to avoid synonymous names for the same compound. The few chemical names not retrieved via the database were manually verified. To determine overlaps with protein-protein interaction databases and facilitate further data integration, the CTD protein names were mapped to the corresponding Ensembl IDs [58] as of June 26, 2008. Only 1.5% of the 42,194 chemical-protein associations could not be clearly identified.

### Structure-target relationship

To investigate chemical space of drugs and environmental compounds, 50 two-dimensional properties were calculated for each structure extracted from PubChem. To visualize them, principal component analysis (PCA) was performed. All necessary data were calculated using the MOE software (Chemical Computing Group version 2007.09)

### Generating a high confidence human Protein-Protein Associations Network

Relevant human chemical-protein associations collected from the CTD were used to create a P-PAN. The maximum number of molecular targets assigned to one compound ‘tert-Butylhydroperoxide’ was 1,189 and the maximum of chemicals assigned to one protein, the cytochrome P450 3A4 (CYP3A4), was 276. The P-PAN was generated by instantiating a node for each protein, and linking by an edge any protein-protein pair where at least one overlapping chemical was identified. Scripts were used to convert the protein-protein associations into a non-redundant list of associations. If proteins A and B are associated, the network may have two associations, A–B and B-A. Only one of these associations was retained in the P-PAN. We assigned two reliability scores to each protein-protein association: a score based on hypergeometric calculation and a weighted score. The weighted score was calculated as the sum of weights for overlapping compounds, where weights were inversely proportional to the number of assigned proteins. The resulting P-PAN is a complex structure containing a total of 2.44 million unique associations between 6,060 human proteins.

### Validating the protein-protein association score

The reliability of the weighted score was confirmed by fitting a calibration curve of different scores against Lage's PPIs^18^ (version 2.9) and Vidal's PPIs^19^. Only 35,000 high confidence experimental interactions were extracted from Lage's PPI, which contains interactions present in the largest databases (Reactome, KEGG…) and data inferred from model organisms. Vidal's PPIs are based on an internal consistent single data source defined using yeast two-hybrid system and contains 3111 interactions.The overlaps of our P-PAN scores and Lage/Vidal PPIs are shown in Figure S2. The benchmark revealed that the weighted score is superior to a score calculated as the negative logarithm of p-values from a test in hypergeometric distribution and a simple overlap count. To estimate the robustness of the model, four thresholds selected from the ‘weighted score’ curves (5%, 8%, 12.5% and 17%) of the complete P-PAN were used to perform prediction for DEHP. At 5%, 73,000 associations between 2105 proteins were extracted. The number of proteins is relatively stable at 8% and 12.5%. However, the number of associations increased significantly from 200,080 to 306,000 including lower score associations in the output file of prediction. The threshold of 17% corresponds to 415,000 associations between 3894 proteins. All thresholds showed a good prediction with the GABA receptors for DEHP. As the 12% threshold already added some more noise in the prediction, we decided to not include more proteins, in order to keep the most significant associations. We then considered a threshold of 8%, represented by the vertical line in Figure S2, which captured a good overlap between our P-PAN and the PPI networks. This selection represents 200,080 associations of the complete P-PAN.

Among the ∼200,000 high confidence associations selected, 3,528 proteins were identified, and these were significantly enriched among the high scoring protein-protein associations as shown in Figure S2 (861 Lage's PPI interactions corresponding to 24.4% were found among the top 5% of the high scoring protein-protein associations). By comparison, only 1,852 of the high confident interactions from Lage were identified in a random P-PAN created by node permutation, and no enrichment was seen for the random network. As example, the selection of high confidence associations allowed to conserve only 803 proteins from the 1189 proteins assigned to the ‘tert-Butylhydroperoxide’.

### P-PAN clustering

A high confidence sub-network of ∼200,000 protein-protein associations was selected which contained 3,528 proteins. This sub-network was highly interconnected, with the majority of proteins belonging to a single large cluster. In order to increase the resolution and facilitate biological interpretation, two clustering methods were applied to the sub-network, MCODE [21] and MCL [22]. We used the default settings for MCODE (fluff option set to 0.1, mode score cutoff set to 0.2, degree cutoff set to 2), and obtained 35 clusters. One major drawback of this algorithm is that not all the proteins in the network were clustered. We used the MCL algorithm with scheme and granularity parameters set to 7 for highest performance and granularity. With the MCL approach we identified a total of 58 clusters as strongly interconnected, with a minimum size of 5 proteins. These clusters were linked together into a new network consisting of a scored cluster-cluster association network. The association score between each cluster pair was calculated from the mean of the P-PAN between each pair of clusters. Each cluster was investigated for functional analysis based on the three Gene Ontology categories (a) molecular function, (b) biological processes, and (c) cellular components as of January 2009. To reduce the noise and improve the quality of the functional annotation, we only used the functional annotation if it was experimentally supported or had traceable references. The following GO evidence codes were allowed: IMP (Inferred from Mutant Phenotype), IGI (Interfered from Genetic Interaction), IPI (Inferred from Physical Interactions) and IDA (Inferred from Direct Assay) and TAS (Traceable Author Statement). At time of use the molecular function category contained 5,981 proteins, the biological processes category 5,196 proteins, and the cellular components 5,151 proteins. We compared human proteins present in GO categories with proteins extracted from the CTD; 14.3% of the CTD proteins could not be annotated for the molecular function, 16.6% for biological processes and 14.9% for cellular components.

To identify chemicals associated with disease, protein-specific information such as involvement in disease was integrated in each cluster. The Online Mendelian Inheritance in Man database (OMIM) [59] (July, 2009) and the GeneCards database [25] (February, 2008) were considered as sources of protein-disease connections. Various clusters were investigated. For example, cluster 1 contained 462 proteins. Using GeneCards, 269 proteins were retrieved with disease annotations. Amongst these 269 proteins, 128 were associated to breast cancer (with give a p-value of 9.67e-18 for breast cancer to cluster 1). Using OMIM, only 90 proteins among the 462 were retrieved with disease annotations. Looking at the cluster enrichment with OMIM, we obtained at the top a non significant p-value of 0.0048 (corresponding to two proteins for paget disease of bone). As another example, we analyzed the second cluster. Cluster 2 contained 433 proteins. 281 proteins were annotated to diseases in Genecards, for only 78 proteins in OMIM. Additionally, cluster 2 has a significant p-value of 2.26e-12 using GeneCards information for necrosis. According to these results we decided to use GeneCards as a source of protein-disease relationships. To avoid too many false positive from Genecards, we set a significance cut-off value of the GeneCards-AKS2 score based on a comparison with OMIM. This was done by overlapping common protein-disease associations from Genecards against OMIM (see Figure S4). The protein-disease connections were kept with a minimum AKS2 score of 60 and p-values were calculated for each disease present in clusters. Then, chemical information from the CTD was integrated with each cluster and p-values were assigned to each chemical. All p-values obtained were calculated using hypergeometric testing, and were corrected for multiple testing with Bonferroni correction [60]. The significance cutoff for the corrected p-values was set to 0.05.

### Neighbor protein procedure

To predict molecular targets for a chemical, a network-neighbor's pull down was done in a three steps procedure: (1) Selection of the input protein(s): Extraction of the protein(s) known to be associated with the selected chemical from the CTD. (2) Identification of network(s) surrounding the input proteins by a neighbor proteins procedure. In this procedure, our P-PAN was queried for the input proteins, and associations between these were added. Next, the first order interactors of all the input proteins were queried and added. For each neighbor, a score was calculated taking into account the topology of the surrounding network, based on the ratio between total associations and associations with input proteins. Molecular targets with a score higher than the threshold (0.1) were kept in the final sub-network(s). This node inclusion parameter is in the conservative end of the optimal range for protein-protein interaction networks^18^. As a final step all proteins in the complex were checked for associations among them and the missing one were added. (3) Establishment of a confidence score for the surrounding network (cscore) and of a score for each protein (cpscore): Each of the pulled down complexes was tested for enrichment on our input set by comparing them against 1.0e4 random complexes for the protein-protein association set to establish a cscore for each sub-network and a cpscore for each connected proteins. The cpscore was used to rank proteins to select potential molecular targets for chemicals. An illustration of cpscore is available on Table S2 for approved drugs.

### Postscript

All the CTD human protein-chemical associations were extracted from the CTD on June 26, 2008. Subsequent updates of CTD, as of June 25, 2009, did not change the overall trends or conclusions of the present study.

## Supporting Information

Figure S1 Structure-target relationship: Oral bioavailability profiles.For drugs, permeability and absorption are properties considered to be important for effective delivery systems, and they receive special attention in pharmaceutical research. We chose to focus on the oral bioavailability properties based on standard Lipinski and Veber rules. It is important to keep in mind that the rules serve as guidelines only - some classes of chemicals, like antibiotics, do not respect the rules. The selected properties are the molecular weight, the octanol/water partition coefficient (an indication of the ability of a molecule to cross biological membranes), the number of hydrogen bond-donor, the number of hydrogen bond-acceptor and the number of rotatable bond. The distributions of the different molecular properties have partial overlaps indicating that small environmental molecules could mimic drug properties. As an example, the distribution of the molecular weight shows a similar profile for each of the three MeSH categories, with a light tendency for ‘Toxic Actions’ chemicals to have a smaller molecular weight (MW). The mean of MW for ‘Toxic Actions’ is 264 daltons whereas the mean of MW for ‘Pharmaceutical Actions’ chemicals is 386 daltons.
(0.06 MB DOC)

Figure S2 Comparing overlaps between protein-protein associations and protein-protein interactions. To assess the reliability of our protein-protein association scores, we fitted a calibration curve of the different PPA scores against overlaps with two PPI databases: the Vidal's interactome and a highly confident set from Lage et al. Vidal's PPIs are based on an internal consistent single data source defined using yeast two-hybrid system. Lage's PPIs contain interactions present in the largest databases and data inferred from model organisms. All the interactions used from Lage et al for the calibration curve are experimental (extracted from Reactome, KEGG and experimental data from small scale experiments). In both comparison, the weighted score (wscore, in red) appears to be superior compared to the score derivates from a hypergeometric test (hscore, in green) and to the random scores. The vertical line represent the threshold selected, which correspond to 8% of the complete P-PAN i.e. 200,080 proteins.
(0.07 MB DOC)

Figure S3 Molecular target predictions for DEHP: novelty of the P-PAN. The novelty of our P-PAN is supported by comparing the predicted proteins associated to DEHP using our approach and an existing method String [1]. Blue nodes are the 15 input proteins known to be associated to this chemical in CTD, green nodes are the predicted proteins from String. Purple nodes are the proteins predicted for DEHP using our P-PAN (dark purple are the proteins with a high confidence score). Green edges are the protein-protein interactions predicted from the String database and purple edges are the protein-protein associations suggested by P-PAN. In the String output network none of the GABA receptors were found, which were identified as potential molecular targets for DEHP using our P-PAN. Considering high confidence score for both methods (String score>0.98), no overlaps between predicted proteins were found. The interactions between predicted proteins were removed for more clarity.
(0.26 MB DOC)

Figure S4 Distributions of the gene- disease scores from GeneCards-AKS2 and OMIN. To integrate disease information to the clusters, GeneCards was used as a source of disease-protein connections. In order to limit the use of false positives present in GeneCards, we mapped shared protein-disease association from OMIN and GeneCards. According to the overlap curves, we set a significant cut-off value of the GeneCards-AKS2 score (in red) of 60.
(0.28 MB DOC)

Text S1 Mining the P-PAN for chemicals associated with diseases.
(0.06 MB DOC)

Text S2 Molecular targets predictions for chemicals.
(0.07 MB DOC)

Table S1 Example of molecular target predictions for chemicals. References: 1. Mahgoub AA, El-Medany AH (2001) Evaluation of chronic exposure of the male rat reproductive system to the insecticide methomyl. Pharmacol. Res. 44:73–80. 2. Bernard L, Martinat N, Lécureuil C, Crépieux P, Reiter E, Tilloy-Ellul A, Chevalier S, Guillou F (2007) Dichlorodiphenyltrichloroethane impairs follicle-stimulating hormone receptor-mediated signaling in rat Sertoli cells. Reprod. Toxicol. 23:158–164. 3. Saqib TA, Naqvi SN, Siddiqui PA, Azmi MA (2005) Detection of pesticide residues in muscles liver and fat of 3 species of Labeo found in Kalri and Haleji lakes. J. Environ. Biol. 26:433–438. 4. Flodström S, Hemming H, Warngard L, Ahlborg UG (1990) Promotion of altered hepatic foci development in rat liver cytochrome P450 enzyme induction and inhibition of cell-cell communication by DDT and some structurally related organohalogen pesticides. Carcinogenesis 11:1413–1417. 5. Sakai H, Iwata H, Kim EY, Tsydenova O, Miyazaki N, Petrov EA, Batoev VB, Tanabe S (2006) Constitutive androstane receptor (CAR) as a potential sensing biomarker of persistent organic pollutants (POPs) in aquatic mammal: molecular characterization expression level and ligand profiling in Baikal seal (Pusa sibirica). Toxicol. Sci. 94:57–70 6. Ding X, Staudinger JL (2005) Repression of PXR-mediated induction of hepatic CYP3A gene expression by protein kinase C. Biochem. Pharmacol. 69:867–873. 7. Matsuura I, Saitoh T, Tani E, Wako Y, Iwata H, Toyota N, Ishizuka Y, Namiki M, Hoshino N, Tsuchitani M, Ikeda Y (2005) Evaluation of a two-generation reproduction toxicity study adding endpoints to detect endocrine disrupting activity using lindane. J. Toxicol. Sci. Spec No 135–161.
(0.04 MB DOC)

Table S2 Illustration of cpscore for approved drugs.
(0.09 MB DOC)
