# Supplementary material for: Deciphering Diseases and Biological Targets for Environmental Chemicals using Toxicogenomics Networks
Source: PLoS Comput Biol. 2010 May 20;6(5):e1000788. doi: 10.1371/journal.pcbi.1000788 (PMC2873901; doi:10.1371/journal.pcbi.1000788)
Supplement: Figure S1 — Structure-target relationship: Oral bioavailability profiles.For drugs, permeability and absorption are properties considered to be important for effective delivery systems, and they receive special attention in pharmaceutical research. We chose to focus on the oral bioavailability properties based on standard Lipinski and Veber rules. It is important to keep in mind that the rules serve as guidelines only - some classes of chemicals, like antibiotics, do not respect the rules. The selected properties are the molecular weight, the octanol/water partition coefficient (an indication of the ability of a molecule to cross biological membranes), the number of hydrogen bond-donor, the number of hydrogen bond-acceptor and the number of rotatable bond. The distributions of the different molecular properties have partial overlaps indicating that small environmental molecules could mimic drug properties. As an example, the distribution of the molecular weight shows a similar profile for each of the three MeSH categories, with a light tendency for ‘Toxic Actions’ chemicals to have a smaller molecular weight (MW). The mean of MW for ‘Toxic Actions’ is 264 daltons whereas the mean of MW for ‘Pharmaceutical Actions’ chemicals is 386 daltons. (0.06 MB DOC) [file pcbi.1000788.s001.doc]

**Structure-target relationship: Oral bioavailability profiles.**


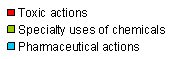


For drugs, permeability and absorption are properties considered to be important for effective delivery systems, and they receive special attention in pharmaceutical research. We chose to focus on the oral bioavailability properties based on standard Lipinski and Veber rules. It is important to keep in mind that the rules serve as guidelines only - some classes of chemicals, like antibiotics, do not respect the rules. The selected properties are the molecular weight, the octanol/water partition coefficient (an indication of the ability of a molecule to cross biological membranes), the number of hydrogen bond-donor, the number of hydrogen bond-acceptor and the number of rotatable bond. The distributions of the different molecular properties have partial overlaps indicating that small environmental molecules could mimic drug properties. As an example, the distribution of the molecular weight shows a similar profile for each of the three MeSH categories, with a light tendency for ‘Toxic Actions’ chemicals to have a smaller molecular weight (MW). The mean of MW for ‘Toxic Actions’ is 264 daltons whereas the mean of MW for ‘Pharmaceutical Actions’ chemicals is 386 daltons.
