# Supplementary material for: Deciphering Diseases and Biological Targets for Environmental Chemicals using Toxicogenomics Networks
Source: PLoS Comput Biol. 2010 May 20;6(5):e1000788. doi: 10.1371/journal.pcbi.1000788 (PMC2873901; doi:10.1371/journal.pcbi.1000788)
Supplement: Figure S2 — Comparing overlaps between protein-protein associations and protein-protein interactions. To assess the reliability of our protein-protein association scores, we fitted a calibration curve of the different PPA scores against overlaps with two PPI databases: the Vidal's interactome and a highly confident set from Lage et al. Vidal's PPIs are based on an internal consistent single data source defined using yeast two-hybrid system. Lage's PPIs contain interactions present in the largest databases and data inferred from model organisms. All the interactions used from Lage et al for the calibration curve are experimental (extracted from Reactome, KEGG and experimental data from small scale experiments). In both comparison, the weighted score (wscore, in red) appears to be superior compared to the score derivates from a hypergeometric test (hscore, in green) and to the random scores. The vertical line represent the threshold selected, which correspond to 8% of the complete P-PAN i.e. 200,080 proteins. (0.07 MB DOC) [file pcbi.1000788.s002.doc]

**Comparing overlaps between protein-protein associations and protein-protein interactions.**

To assess the reliability of our protein-protein association scores, we fitted a calibration curve of the different PPA scores against overlaps with two PPI databases: the Vidal´s interactome and a highly confident set from Lage *et al*. Vidal´s PPIs are based on an internal consistent single data source defined using yeast two-hybrid system. Lage´s PPIs contain interactions present in the largest databases and data inferred from model organisms. All the interactions used from Lage *et al* for the calibration curve are experimental (extracted from Reactome, KEGG and experimental data from small scale experiments). In both comparison, the weighted score (wscore, in red) appears to be superior compared to the score derivates from a hypergeometric test (hscore, in green) and to the random scores. The vertical line represent the threshold selected, which correspond to 8% of the complete P-PAN i.e. 200,080 proteins.
