# Supplementary material for: Deciphering Diseases and Biological Targets for Environmental Chemicals using Toxicogenomics Networks
Source: PLoS Comput Biol. 2010 May 20;6(5):e1000788. doi: 10.1371/journal.pcbi.1000788 (PMC2873901; doi:10.1371/journal.pcbi.1000788)
Supplement: Figure S3 — Molecular target predictions for DEHP: novelty of the P-PAN. The novelty of our P-PAN is supported by comparing the predicted proteins associated to DEHP using our approach and an existing method String [1]. Blue nodes are the 15 input proteins known to be associated to this chemical in CTD, green nodes are the predicted proteins from String. Purple nodes are the proteins predicted for DEHP using our P-PAN (dark purple are the proteins with a high confidence score). Green edges are the protein-protein interactions predicted from the String database and purple edges are the protein-protein associations suggested by P-PAN. In the String output network none of the GABA receptors were found, which were identified as potential molecular targets for DEHP using our P-PAN. Considering high confidence score for both methods (String score>0.98), no overlaps between predicted proteins were found. The interactions between predicted proteins were removed for more clarity. (0.26 MB DOC) [file pcbi.1000788.s003.doc]

**Molecular target predictions for DEHP: novelty of the P-PAN.**

The novelty of our P-PAN is supported by comparing the predicted proteins associated to DEHP using our approach and an existing method String [1]. Blue nodes are the 15 input proteins known to be associated to this chemical in CTD, green nodes are the predicted proteins from String. Purple nodes are the proteins predicted for DEHP using our P-PAN (dark purple are the proteins with a high confidence score). Green edges are the protein-protein interactions predicted from the String database and purple edges are the protein-protein associations suggested by P-PAN. In the String output network none of the GABA receptors were found, which were identified as potential molecular targets for DEHP using our P-PAN. Considering high confidence score for both methods (String score > 0.98), no overlaps between predicted proteins were found. The interactions between predicted proteins were removed for more clarity.

Reference:

1. von Mering C, [Jensen LJ](http://www.ncbi.nlm.nih.gov/sites/entrez?Db=pubmed&Cmd=Search&Term="Jensen LJ"%5BAuthor%5D&itool=EntrezSystem2.PEntrez.Pubmed.Pubmed_ResultsPanel.Pubmed_DiscoveryPanel.Pubmed_RVAbstractPlus), [Kuhn M](http://www.ncbi.nlm.nih.gov/sites/entrez?Db=pubmed&Cmd=Search&Term="Kuhn M"%5BAuthor%5D&itool=EntrezSystem2.PEntrez.Pubmed.Pubmed_ResultsPanel.Pubmed_DiscoveryPanel.Pubmed_RVAbstractPlus), [Chaffron S](http://www.ncbi.nlm.nih.gov/sites/entrez?Db=pubmed&Cmd=Search&Term="Chaffron S"%5BAuthor%5D&itool=EntrezSystem2.PEntrez.Pubmed.Pubmed_ResultsPanel.Pubmed_DiscoveryPanel.Pubmed_RVAbstractPlus), [Doerks T](http://www.ncbi.nlm.nih.gov/sites/entrez?Db=pubmed&Cmd=Search&Term="Doerks T"%5BAuthor%5D&itool=EntrezSystem2.PEntrez.Pubmed.Pubmed_ResultsPanel.Pubmed_DiscoveryPanel.Pubmed_RVAbstractPlus) et al. (2007) STRING 7-- recent developments in the integration and prediction of protein interactions. *Nucleic Acids Res.* **35**:358-362
