# Supplementary material for: Deciphering Diseases and Biological Targets for Environmental Chemicals using Toxicogenomics Networks
Source: PLoS Comput Biol. 2010 May 20;6(5):e1000788. doi: 10.1371/journal.pcbi.1000788 (PMC2873901; doi:10.1371/journal.pcbi.1000788)
Supplement: Figure S4 — Distributions of the gene- disease scores from GeneCards-AKS2 and OMIN. To integrate disease information to the clusters, GeneCards was used as a source of disease-protein connections. In order to limit the use of false positives present in GeneCards, we mapped shared protein-disease association from OMIN and GeneCards. According to the overlap curves, we set a significant cut-off value of the GeneCards-AKS2 score (in red) of 60. (0.28 MB DOC) [file pcbi.1000788.s004.doc]

**Distributions of the gene- disease scores from GeneCards-AKS2 and OMIN.**


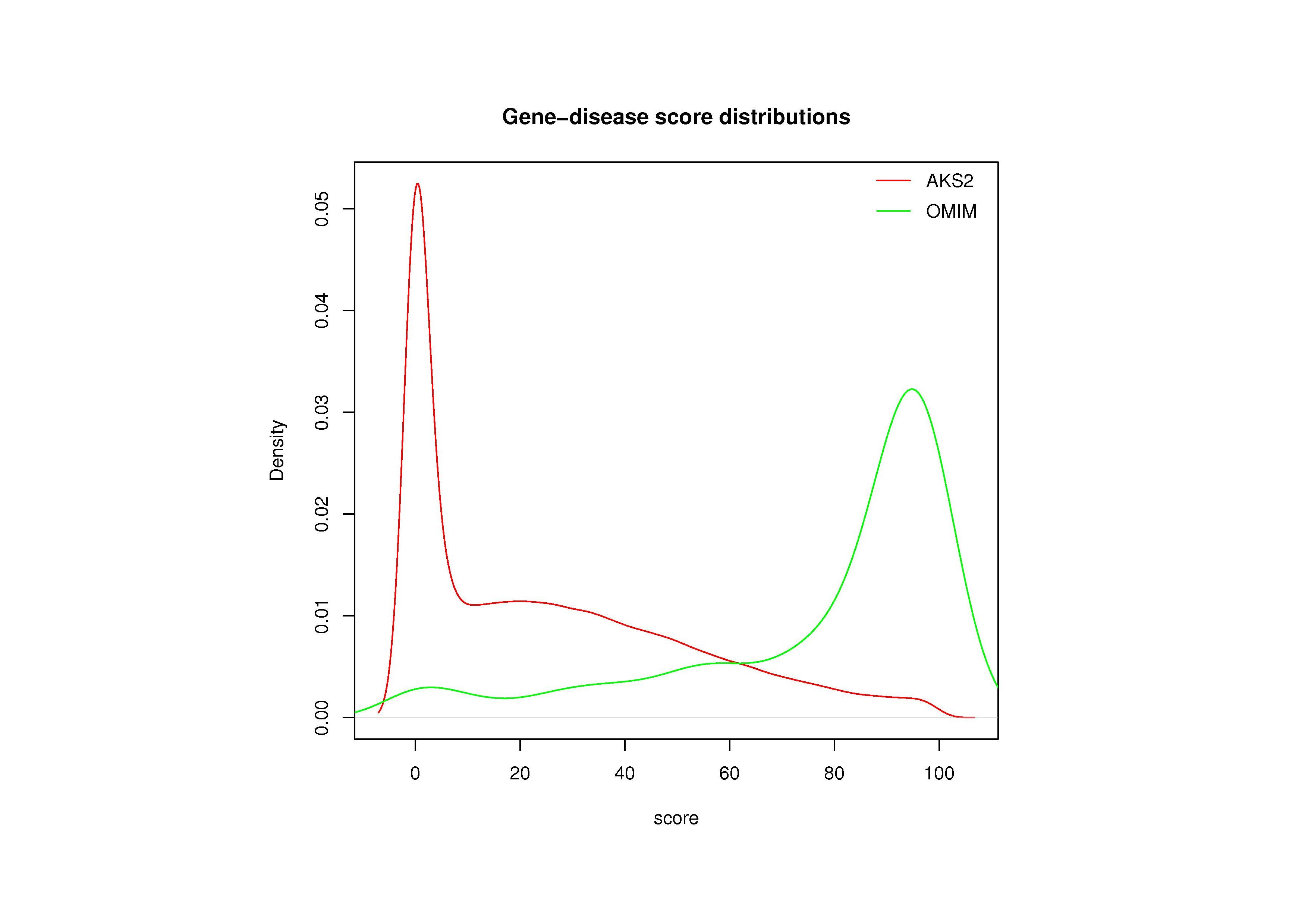


To integrate disease information to the clusters, GeneCards was used as a source of disease-protein connections. In order to limit the use of false positives present in GeneCards, we mapped shared protein-disease association from OMIN and GeneCards. According to the overlap curves, we set a significant cut-off value of the GeneCards-AKS2 score (in red) of 60.
