# Supplementary material for: Deciphering Diseases and Biological Targets for Environmental Chemicals using Toxicogenomics Networks
Source: PLoS Comput Biol. 2010 May 20;6(5):e1000788. doi: 10.1371/journal.pcbi.1000788 (PMC2873901; doi:10.1371/journal.pcbi.1000788)
Supplement: Text S1 — Mining the P-PAN for chemicals associated with diseases. (0.06 MB DOC) [file pcbi.1000788.s005.doc]

**Mining the P-PAN for chemicals associated with diseases**

We will here provide more details on the few selected clusters. The cluster highly enriched for breast cancer, contains 462 genes, which are associated to steroid binding (GO molecular function) and DNA replication (GO biological process). Among the chemicals potentially associated to breast cancer, estradiol is the most significantly associated chemical. Interestingly, patients with a past history of breast cancer are not recommended to use estradiol or estrone as estrogen supplementation due to DNA-damaging effects of estrogens [1]. The second highest associated chemical is bisphenol A. Animal studies on low dose exposure of bisphenol A, a chemical used in production of polycarbonate plastic with low acute toxicity, show changes in breast tissue that predispose cells to hormones and carcinogens [2-3]. A third chemical, genistein, a soy product, phyto-estrogen, is known from literature to act as both an estrogen and an antiproliferative agent [4]. A recent study demonstrates that genistein could contribute to some specific forms of breast cancer, by stimulating growth of human breast cancer cells in a postmenopausal animal model, with low plasma estradiol concentrations [5]. As a last example of a highly associated chemical to the breast cancer disease we will mention fluvestrant. Fluvestrant is a drug used in hormonal therapy for the treatment of breast cancer in women who have had their change of life (menopause). All these significantly associated chemicals to breast cancer are already known from the literature, thus supporting the clustering quality of the P-PAN.

The cluster enriched for lung cancer contains 59 genes and is involved in glutathione transferase (GST) activity according to GO molecular function enrichment. The link between GST activity and lung cancer is already known [6]. Styrene, a suspected chemical linked to cancer diseases, is associated to the cluster. Styrene is the precursor of polystyrene, an important synthetic materiel. It is classified as a possible human carcinogen by the [International Agency for Research on Cancer](http://en.wikipedia.org/wiki/International_Agency_for_Research_on_Cancer) (IARC) ([Styrene](http://www.osha.gov/SLTC/styrene/index.html). [Occupational Safety and Health Administration](http://en.wikipedia.org/wiki/Occupational_Safety_and_Health_Administration): www.osha.gov). The U.S. Environmental Protection Agency (EPA) does not have a cancer classification for styrene, but is evaluating its potential carcinogenicity. The EPA has described styrene as "a suspected carcinogen" and "a suspected toxin to the gastrointestinal, kidney, and respiratory systems making the link to lung cancer, among others. A study shows a possible but very low lung tumorigenic risk of styrene from daily intake via food and ambient air [7]. The last cluster analyzed is associated to necrosis and seems to be enriched in caspase activity (GO molecular function) and regulation of apoptose (GO biological process). It is known that caspases are cysteine proteases which play essential roles in apoptosis, necrosis and inflammation. Regarding chemicals associated to this cluster, realgar is the most significantly associated chemical. Realgar, an arsenic sulfide mineral very poisonous has been used for firework manufacturers. It has been shown in one study to induce apoptosis and necrosis in human leukemia cell lines [8]. The third most significantly associated chemical is doxorubicin, which is a drug widely used in cancer chemotherapy. Our approach, i.e. the proposed method based on P-PAN, shows its capability of recovering known chemical-disease associations as well as suggesting interesting links between chemical and disease.

References:

1. Cavalieri E, [Frenkel K](http://www.ncbi.nlm.nih.gov/sites/entrez?Db=pubmed&Cmd=Search&Term="Frenkel K"%5BAuthor%5D&itool=EntrezSystem2.PEntrez.Pubmed.Pubmed_ResultsPanel.Pubmed_DiscoveryPanel.Pubmed_RVAbstractPlus), [Liehr JG](http://www.ncbi.nlm.nih.gov/sites/entrez?Db=pubmed&Cmd=Search&Term="Liehr JG"%5BAuthor%5D&itool=EntrezSystem2.PEntrez.Pubmed.Pubmed_ResultsPanel.Pubmed_DiscoveryPanel.Pubmed_RVAbstractPlus), [Rogan E](http://www.ncbi.nlm.nih.gov/sites/entrez?Db=pubmed&Cmd=Search&Term="Rogan E"%5BAuthor%5D&itool=EntrezSystem2.PEntrez.Pubmed.Pubmed_ResultsPanel.Pubmed_DiscoveryPanel.Pubmed_RVAbstractPlus), [Roy D](http://www.ncbi.nlm.nih.gov/sites/entrez?Db=pubmed&Cmd=Search&Term="Roy D"%5BAuthor%5D&itool=EntrezSystem2.PEntrez.Pubmed.Pubmed_ResultsPanel.Pubmed_DiscoveryPanel.Pubmed_RVAbstractPlus) (2000) Estrogens as Endogenous Genotoxic Agents-DNA adducts and mutations. *J. Natl. Cancer Inst. Monogr.* 27:75-94.

2. Muñoz-de-Toro M, [Markey CM](http://www.ncbi.nlm.nih.gov/sites/entrez?Db=pubmed&Cmd=Search&Term="Markey CM"%5BAuthor%5D&itool=EntrezSystem2.PEntrez.Pubmed.Pubmed_ResultsPanel.Pubmed_DiscoveryPanel.Pubmed_RVAbstractPlus), [Wadia PR](http://www.ncbi.nlm.nih.gov/sites/entrez?Db=pubmed&Cmd=Search&Term="Wadia PR"%5BAuthor%5D&itool=EntrezSystem2.PEntrez.Pubmed.Pubmed_ResultsPanel.Pubmed_DiscoveryPanel.Pubmed_RVAbstractPlus), [Luque EH](http://www.ncbi.nlm.nih.gov/sites/entrez?Db=pubmed&Cmd=Search&Term="Luque EH"%5BAuthor%5D&itool=EntrezSystem2.PEntrez.Pubmed.Pubmed_ResultsPanel.Pubmed_DiscoveryPanel.Pubmed_RVAbstractPlus), [Rubin BS](http://www.ncbi.nlm.nih.gov/sites/entrez?Db=pubmed&Cmd=Search&Term="Rubin BS"%5BAuthor%5D&itool=EntrezSystem2.PEntrez.Pubmed.Pubmed_ResultsPanel.Pubmed_DiscoveryPanel.Pubmed_RVAbstractPlus) et al. (2005) [Perinatal exposure to bisphenol-A alters peripubertal mammary gland development in mice](http://endo.endojournals.org/cgi/pmidlookup?view=long&pmid=15919749). [*Endocrinology*](http://en.wikipedia.org/wiki/Endocrinology) 146:4138–4147.

3. Murray TJ, [Maffini MV](http://www.ncbi.nlm.nih.gov/sites/entrez?Db=pubmed&Cmd=Search&Term="Maffini MV"%5BAuthor%5D&itool=EntrezSystem2.PEntrez.Pubmed.Pubmed_ResultsPanel.Pubmed_DiscoveryPanel.Pubmed_RVAbstractPlus), [Ucci AA](http://www.ncbi.nlm.nih.gov/sites/entrez?Db=pubmed&Cmd=Search&Term="Ucci AA"%5BAuthor%5D&itool=EntrezSystem2.PEntrez.Pubmed.Pubmed_ResultsPanel.Pubmed_DiscoveryPanel.Pubmed_RVAbstractPlus), [Sonnenschein C](http://www.ncbi.nlm.nih.gov/sites/entrez?Db=pubmed&Cmd=Search&Term="Sonnenschein C"%5BAuthor%5D&itool=EntrezSystem2.PEntrez.Pubmed.Pubmed_ResultsPanel.Pubmed_DiscoveryPanel.Pubmed_RVAbstractPlus), [Soto AM](http://www.ncbi.nlm.nih.gov/sites/entrez?Db=pubmed&Cmd=Search&Term="Soto AM"%5BAuthor%5D&itool=EntrezSystem2.PEntrez.Pubmed.Pubmed_ResultsPanel.Pubmed_DiscoveryPanel.Pubmed_RVAbstractPlus) (2007) [Induction of mammary gland ductal hyperplasias and carcinoma in situ following fetal bisphenol A exposure](http://linkinghub.elsevier.com/retrieve/pii/S0890-6238(06)00263-2). [*Reprod. Toxicol*](http://en.wikipedia.org/w/index.php?title=Reprod._Toxicol.&action=edit&redlink=1)*.* 23:383–390.

4. Bouker KB, Hilakivi-Clarke L (2000) Genistein: Does it prevent or promote breast cancer? *Environ. health perspect.* 108:701-708.

5. Ju YH, Allred KF, Allred CD, Helferich WG (2006) Genistein stimulates growth of human breast cancer cells in a novel postmenopausal animal model with low plasma estradiol concentrations. *Carcinogenesis* 27:1292-1299.

6. Carlsten C, [Sagoo GS](http://www.ncbi.nlm.nih.gov/sites/entrez?Db=pubmed&Cmd=Search&Term="Sagoo GS"%5BAuthor%5D&itool=EntrezSystem2.PEntrez.Pubmed.Pubmed_ResultsPanel.Pubmed_DiscoveryPanel.Pubmed_RVAbstractPlus), [Frodsham AJ](http://www.ncbi.nlm.nih.gov/sites/entrez?Db=pubmed&Cmd=Search&Term="Frodsham AJ"%5BAuthor%5D&itool=EntrezSystem2.PEntrez.Pubmed.Pubmed_ResultsPanel.Pubmed_DiscoveryPanel.Pubmed_RVAbstractPlus), [Burke W](http://www.ncbi.nlm.nih.gov/sites/entrez?Db=pubmed&Cmd=Search&Term="Burke W"%5BAuthor%5D&itool=EntrezSystem2.PEntrez.Pubmed.Pubmed_ResultsPanel.Pubmed_DiscoveryPanel.Pubmed_RVAbstractPlus), [Higgins JP](http://www.ncbi.nlm.nih.gov/sites/entrez?Db=pubmed&Cmd=Search&Term="Higgins JP"%5BAuthor%5D&itool=EntrezSystem2.PEntrez.Pubmed.Pubmed_ResultsPanel.Pubmed_DiscoveryPanel.Pubmed_RVAbstractPlus) (2008) Glutathione S-transferase M1 (GSTM1) polymorphisms and lung cancer: a literature-based systematic HuGE review and meta-analysis. *Am. J. Epidemiol.* 167:759-774.

7. [Filser JG](http://www.ncbi.nlm.nih.gov/sites/entrez?Db=pubmed&Cmd=Search&Term="Filser JG"%5BAuthor%5D&itool=EntrezSystem2.PEntrez.Pubmed.Pubmed_ResultsPanel.Pubmed_RVAbstract), Kessler W, Csanady GA (2002) Estimation of a possible tumorigenic risk of styrene from daily intake via food and ambient air. [*Toxicol. Lett*](javascript:AL_get(this, 'jour', 'Toxicol Lett.');)*.* 126:1-18 .

8. [Ning N](http://www.ncbi.nlm.nih.gov/sites/entrez?Db=pubmed&Cmd=Search&Term="Ning N"%5BAuthor%5D&itool=EntrezSystem2.PEntrez.Pubmed.Pubmed_ResultsPanel.Pubmed_DiscoveryPanel.Pubmed_RVAbstractPlus), [Peng ZF](http://www.ncbi.nlm.nih.gov/sites/entrez?Db=pubmed&Cmd=Search&Term="Peng ZF"%5BAuthor%5D&itool=EntrezSystem2.PEntrez.Pubmed.Pubmed_ResultsPanel.Pubmed_DiscoveryPanel.Pubmed_RVAbstractPlus), [Yuan L](http://www.ncbi.nlm.nih.gov/sites/entrez?Db=pubmed&Cmd=Search&Term="Yuan L"%5BAuthor%5D&itool=EntrezSystem2.PEntrez.Pubmed.Pubmed_ResultsPanel.Pubmed_DiscoveryPanel.Pubmed_RVAbstractPlus), [Gou BD](http://www.ncbi.nlm.nih.gov/sites/entrez?Db=pubmed&Cmd=Search&Term="Gou BD"%5BAuthor%5D&itool=EntrezSystem2.PEntrez.Pubmed.Pubmed_ResultsPanel.Pubmed_DiscoveryPanel.Pubmed_RVAbstractPlus), [Zhang TL](http://www.ncbi.nlm.nih.gov/sites/entrez?Db=pubmed&Cmd=Search&Term="Zhang TL"%5BAuthor%5D&itool=EntrezSystem2.PEntrez.Pubmed.Pubmed_ResultsPanel.Pubmed_DiscoveryPanel.Pubmed_RVAbstractPlus) et al. (2005) Realgar nano-particles induce apoptosis and necrosis in leukemia cell lines K562 and HL-60. [*Zhongguo Zhong Yao Za Zhi*](javascript:AL_get(this, 'jour', 'Zhongguo Zhong Yao Za Zhi.');) 30:136-140.
