# Supplementary material for: Deciphering Diseases and Biological Targets for Environmental Chemicals using Toxicogenomics Networks
Source: PLoS Comput Biol. 2010 May 20;6(5):e1000788. doi: 10.1371/journal.pcbi.1000788 (PMC2873901; doi:10.1371/journal.pcbi.1000788)
Supplement: Text S2 — Molecular targets predictions for chemicals. (0.07 MB DOC) [file pcbi.1000788.s006.doc]

**Molecular targets predictions for chemicals.**

We will here provide more details on the chemicals selected in the main text and their potential molecular targets –known or novel- suggested by our method.

We used the 15 proteins associated to DEHP in CTD to query the network by our neighbor protein procedure approach (see Material and Methods). A complex of proteins was identified with a confidence score (cscore) of 26.56. For DEHP among the six highly ranked predicted proteins, adrenocorticotropin (POMC) is a new potential target. This prediction is supported by Supornislchai *et al* [1] which have demonstrated that exposure of male rats to DEHP leads to an age-dependant activation of POMC based on in vivo and in vitro experimental data. From translational perspectives, their finding may have implications for humans being and in particular for newborns and children who may be more sensitive to environmental levels of DEHP than adults. Studies show that neonates are often more receptive than adults to the toxic effects of chemicals [2] and younger rats studies confirmed that they are more sensitive than adults to DEHP exposure [3]. POMC is a target known to be associated with Cushing syndrome (hormonal disorder caused by prolonged exposure of the body´s tissues to high level of hormone e.g. cortisol). People with Cushing syndrome have tendency to develop tumors of one or more endocrine glands. POMC is also link for example to ACTH syndrome (complex syndrome due to adrenocorticotrophic hormone, ATCH, production by non-pituitary neoplasms e.g. lung cancer). Another potential target shown for DEHP is the cytochrome P450 3A11 (CYP3A11). This association is supported by Fan *et al* [4] where their studies demonstrate that DEHP increased CYP3A11 in mice. CYP3A11 is a protein involved in the metabolism of xenobiotics and thus maybe involved in the transformation of DEHP to its mono metabolite MEHP, which is known to be toxic.

Regarding TCDD, a complex of proteins was identified with a cscore of 103.85. Among the most significant proteins predicted, six high ranked proteins are not referred in the CTD input dataset as TCDD targets. Five of these six new potential targets are supported by literature, which supported the high quality of the network. A study showed an association between PRKCE, protein kinase C elipson, and TCDD [5]. TCDD binds to the aryl hydrocarbon receptor (AHR), which affects the localization of PRKCE protein. This finding may contribute to our understanding of the mechanism of TCDD-related neurotoxicity, and thereby improving the health risk assessment of neurotoxic compounds in humans. PRKCE is a protein known to be involved in primary CNS tumors, astrocytoma, and brain tumors. The second potential associated target to TCDD is POMC. Studies have shown that TCDD results in increased expression of POMC mRNA on mouse [6] and rat [7]. Another predicted target, CPT1A, which is the carnitine palmitoyltransferase 1A, is supported by gene expressions analyses. TCDD results in increased expression of CPT1A mRNA in rat. CPT1A is involved in hypoglycemia and obesity. The next target predicted to be associated with TCDD is MPV, major vault protein. This protein is a lung resistance related protein which mediated drug resistance, involved in leukemia. No support from literature has been found. Boverhoh *et al* [8] shown in one study that TCDD results in decreased expression of HSD11B1 mRNA, hydroxysteroid dehydrogenase. HSD11B1 is linked to obesity, cushing and ach syndromes and metabolic disorders. The last target prediction is APOB, apolipoprotein B. TCDD has been shown in decreased expression of APOB mRNA in Japanese fish [9]. APOB is known to be involved in hypercholesterolemia and obesity.

For the drug pirinixic acid (PA), a complex of proteins was identified with a cscore of 12.59. Among then, five high ranked proteins, not referred in the CTD input dataset as PA targets are supported by literature. A recent study from 2008 on hepatic gene expression in mice shown that PA results in increased expression of ADRA1A mRNA, ADRA2A mRNA, which are adrenergic, alpha-1A/2A-receptors; of ALDH3A2, aldehyde dehydrogenase 3 family; and of CST3, cysteine proteinase inhibitor [10]. Attention-deficit and hyperactivity disorder have been link to ADRA1A and ADRA2A proteins. A patent application (WO/2003/075911) suggested the use of PA as a medicament for treatment of proliferative vascular disease e.g. atherosclerosis or hypertension. Regarding ALDH3, this protein is involved in breast cancer metastatic and hepatocellular carcinoma. Despite low cpscores, potential association between PA and these genes are supported by literature. Another predicted target, the C3 protein is an acylation stimulating protein. Amacher et al. show that PA affects the expression of C3 protein in rat [11]. C3 is a protein involved in necrosis and afibrinogenemia (blood disorders).

For the last chemical studied, the permethrin, a complex of proteins was identified with cscore of 28.51. Among then, three high ranked proteins, not referred in the CTD input dataset as permethrin targets are supported by literature. The potential association between permethrin and CYP2B1, a cytochrome P450, have been mentioned in several rat studies showing that that permethrin increase expression of CYP2B1 mRNA [12-13]. CYP2B1 is a protein involved in the metabolism of hormone like chemical e.g. testosterone and androstenedione. SHBG, sex hormone-binding globulin, is predicted to be associated with permethrin. A study supports this association indicating that a novel class of non-steroidal compounds, the pyrethroids (e.g. permethrin), can interact competitively with human androgen receptors and SHBG. These findings provide a mechanism by which chronic exposure of humans or animals to pesticides containing these compounds may result in disturbances in endocrine effects relating to androgen action [14]. SHBG is a protein involved in polycystic ovary syndrome, hyperandrogenism and endocrine effects. CYP2B6 is a predicted potential target for permethrin but is not supported by literature. Another protein predicted is NRI13, a nuclear receptor subfamily 1. Permethrin has been shown to increase expression of NRI13mRNA on house mouse study [15]. NRI13 is known to be link to hyperparathyroidism and diverse syndromes associated to thyroid.

References:

1. Supornsilchai V, Söder O, Svechnikov K (2007) Stimulation of the pituitary-adrenal axis and of adrenocortical steroidogenesis ex vivo by administration of di-2ethylhexyl phthalate to prepubertal male rats. *J. Endocrinol.* 192:33-39.

2. Parke DV (1984) Development of detoxification mechanisms in the neonate. In *Toxicology and the New born.* Kacew S Reasor MJ (ed) pp1-31, Elsevier, New York, USA.

3. Dostal LA, Jenkins WL, Schwetz BA (1987) Hepatic peroxisome proliferation and hypolipidemic effects of di(2-ethylhexyl)phthalate in neonatal and adult rats. *Toxic. Appl. Pharmacol.* 87:81-90.

4. Fan LQ, [You L](http://www.ncbi.nlm.nih.gov/sites/entrez?Db=pubmed&Cmd=Search&Term="You L"%5BAuthor%5D&itool=EntrezSystem2.PEntrez.Pubmed.Pubmed_ResultsPanel.Pubmed_DiscoveryPanel.Pubmed_RVAbstractPlus), [Brown-Borg H](http://www.ncbi.nlm.nih.gov/sites/entrez?Db=pubmed&Cmd=Search&Term="Brown-Borg H"%5BAuthor%5D&itool=EntrezSystem2.PEntrez.Pubmed.Pubmed_ResultsPanel.Pubmed_DiscoveryPanel.Pubmed_RVAbstractPlus), [Brown S](http://www.ncbi.nlm.nih.gov/sites/entrez?Db=pubmed&Cmd=Search&Term="Brown S"%5BAuthor%5D&itool=EntrezSystem2.PEntrez.Pubmed.Pubmed_ResultsPanel.Pubmed_DiscoveryPanel.Pubmed_RVAbstractPlus), [Edwards RJ](http://www.ncbi.nlm.nih.gov/sites/entrez?Db=pubmed&Cmd=Search&Term="Edwards RJ"%5BAuthor%5D&itool=EntrezSystem2.PEntrez.Pubmed.Pubmed_ResultsPanel.Pubmed_DiscoveryPanel.Pubmed_RVAbstractPlus) et al. (2004) Regulation of phase I and phase II steroid metabolism enzymes by PPAR alpha activators. *Toxicology* 204:109-121.

5. Kim SY, Yang JH (2005) **Neurotoxic effects of 2378-tetrachlorodibenzo-p-dioxin in cerebellar granule cells.** *Exp. Mol. Med.*  37:58-64.

6. Huang P, [Ceccatelli S](http://www.ncbi.nlm.nih.gov/sites/entrez?Db=pubmed&Cmd=Search&Term="Ceccatelli S"%5BAuthor%5D&itool=EntrezSystem2.PEntrez.Pubmed.Pubmed_ResultsPanel.Pubmed_DiscoveryPanel.Pubmed_RVAbstractPlus), [Håkansson H](http://www.ncbi.nlm.nih.gov/sites/entrez?Db=pubmed&Cmd=Search&Term="Håkansson H"%5BAuthor%5D&itool=EntrezSystem2.PEntrez.Pubmed.Pubmed_ResultsPanel.Pubmed_DiscoveryPanel.Pubmed_RVAbstractPlus), [Grandison L](http://www.ncbi.nlm.nih.gov/sites/entrez?Db=pubmed&Cmd=Search&Term="Grandison L"%5BAuthor%5D&itool=EntrezSystem2.PEntrez.Pubmed.Pubmed_ResultsPanel.Pubmed_DiscoveryPanel.Pubmed_RVAbstractPlus), [Rannug A](http://www.ncbi.nlm.nih.gov/sites/entrez?Db=pubmed&Cmd=Search&Term="Rannug A"%5BAuthor%5D&itool=EntrezSystem2.PEntrez.Pubmed.Pubmed_ResultsPanel.Pubmed_DiscoveryPanel.Pubmed_RVAbstractPlus) (2002) Constitutive and TCDD induced expression of Ah receptor responsive genes in the pituitary. *Neurotoxicology* 23:783-793.

7. Fetissov SO, [Huang P](http://www.ncbi.nlm.nih.gov/sites/entrez?Db=pubmed&Cmd=Search&Term="Huang P"%5BAuthor%5D&itool=EntrezSystem2.PEntrez.Pubmed.Pubmed_ResultsPanel.Pubmed_DiscoveryPanel.Pubmed_RVAbstractPlus), [Zhang Q](http://www.ncbi.nlm.nih.gov/sites/entrez?Db=pubmed&Cmd=Search&Term="Zhang Q"%5BAuthor%5D&itool=EntrezSystem2.PEntrez.Pubmed.Pubmed_ResultsPanel.Pubmed_DiscoveryPanel.Pubmed_RVAbstractPlus), [Mimura J](http://www.ncbi.nlm.nih.gov/sites/entrez?Db=pubmed&Cmd=Search&Term="Mimura J"%5BAuthor%5D&itool=EntrezSystem2.PEntrez.Pubmed.Pubmed_ResultsPanel.Pubmed_DiscoveryPanel.Pubmed_RVAbstractPlus), [Fujii-Kuriyama Y](http://www.ncbi.nlm.nih.gov/sites/entrez?Db=pubmed&Cmd=Search&Term="Fujii-Kuriyama Y"%5BAuthor%5D&itool=EntrezSystem2.PEntrez.Pubmed.Pubmed_ResultsPanel.Pubmed_DiscoveryPanel.Pubmed_RVAbstractPlus) et al. (2004) Expression of hypothalamic neuropeptides after acute TCDD treatment and distribution of Ah receptor repressor. *Regul. Pept.* 119:113-124.

8. Boverhof DR, [Burgoon LD](http://www.ncbi.nlm.nih.gov/sites/entrez?Db=pubmed&Cmd=Search&Term="Burgoon LD"%5BAuthor%5D&itool=EntrezSystem2.PEntrez.Pubmed.Pubmed_ResultsPanel.Pubmed_DiscoveryPanel.Pubmed_RVAbstractPlus), [Tashiro C](http://www.ncbi.nlm.nih.gov/sites/entrez?Db=pubmed&Cmd=Search&Term="Tashiro C"%5BAuthor%5D&itool=EntrezSystem2.PEntrez.Pubmed.Pubmed_ResultsPanel.Pubmed_DiscoveryPanel.Pubmed_RVAbstractPlus), [Sharratt B](http://www.ncbi.nlm.nih.gov/sites/entrez?Db=pubmed&Cmd=Search&Term="Sharratt B"%5BAuthor%5D&itool=EntrezSystem2.PEntrez.Pubmed.Pubmed_ResultsPanel.Pubmed_DiscoveryPanel.Pubmed_RVAbstractPlus), [Chittim B](http://www.ncbi.nlm.nih.gov/sites/entrez?Db=pubmed&Cmd=Search&Term="Chittim B"%5BAuthor%5D&itool=EntrezSystem2.PEntrez.Pubmed.Pubmed_ResultsPanel.Pubmed_DiscoveryPanel.Pubmed_RVAbstractPlus) et al. (2006) **Comparative toxicogenomic analysis of the hepatotoxic effects of TCDD in Sprague Dawley rats and C57BL/6 mice.** *Toxicol. Sci.* 94:398-416.

9. Volz DC, [Bencic DC](http://www.ncbi.nlm.nih.gov/sites/entrez?Db=pubmed&Cmd=Search&Term="Bencic DC"%5BAuthor%5D&itool=EntrezSystem2.PEntrez.Pubmed.Pubmed_ResultsPanel.Pubmed_DiscoveryPanel.Pubmed_RVAbstractPlus), [Hinton DE](http://www.ncbi.nlm.nih.gov/sites/entrez?Db=pubmed&Cmd=Search&Term="Hinton DE"%5BAuthor%5D&itool=EntrezSystem2.PEntrez.Pubmed.Pubmed_ResultsPanel.Pubmed_DiscoveryPanel.Pubmed_RVAbstractPlus), [Law JM](http://www.ncbi.nlm.nih.gov/sites/entrez?Db=pubmed&Cmd=Search&Term="Law JM"%5BAuthor%5D&itool=EntrezSystem2.PEntrez.Pubmed.Pubmed_ResultsPanel.Pubmed_DiscoveryPanel.Pubmed_RVAbstractPlus), [Kullman SW](http://www.ncbi.nlm.nih.gov/sites/entrez?Db=pubmed&Cmd=Search&Term="Kullman SW"%5BAuthor%5D&itool=EntrezSystem2.PEntrez.Pubmed.Pubmed_ResultsPanel.Pubmed_DiscoveryPanel.Pubmed_RVAbstractPlus) (2005) **2,3,7,8-Tetrachlorodibenzo-p-dioxin (TCDD) induces organ- specific differential gene expression in male Japanese medaka (Oryzias latipes).** *Toxicol. Sci.* 85:572-584.

10. Sanderson LM, [de Groot PJ](http://www.ncbi.nlm.nih.gov/sites/entrez?Db=pubmed&Cmd=Search&Term="de Groot PJ"%5BAuthor%5D&itool=EntrezSystem2.PEntrez.Pubmed.Pubmed_ResultsPanel.Pubmed_DiscoveryPanel.Pubmed_RVAbstractPlus), [Hooiveld GJ](http://www.ncbi.nlm.nih.gov/sites/entrez?Db=pubmed&Cmd=Search&Term="Hooiveld GJ"%5BAuthor%5D&itool=EntrezSystem2.PEntrez.Pubmed.Pubmed_ResultsPanel.Pubmed_DiscoveryPanel.Pubmed_RVAbstractPlus), [Koppen A](http://www.ncbi.nlm.nih.gov/sites/entrez?Db=pubmed&Cmd=Search&Term="Koppen A"%5BAuthor%5D&itool=EntrezSystem2.PEntrez.Pubmed.Pubmed_ResultsPanel.Pubmed_DiscoveryPanel.Pubmed_RVAbstractPlus), [Kalkhoven E](http://www.ncbi.nlm.nih.gov/sites/entrez?Db=pubmed&Cmd=Search&Term="Kalkhoven E"%5BAuthor%5D&itool=EntrezSystem2.PEntrez.Pubmed.Pubmed_ResultsPanel.Pubmed_DiscoveryPanel.Pubmed_RVAbstractPlus) et al. (2008) **Effect of synthetic dietary triglycerides: a novel research paradigm for nutrigenomics.** *PLoS ONE* 3(2):e1681.

11. [Amacher DE](http://ctd.mdibl.org/query.go?type=reference&action=search&partyNmQueryType=equals&partyNm=Amacher+DE), Adler R, Herath A, Townsend RR (2005) **Use of proteomic methods to identify serum biomarkers associated with rat liver toxicity or hypertrophy.** *Clin. Chem.* 51:1796-1803.

12. Bauer D, Wolfram N, Kahl GF, Hirsh-Ernst KI (2004) **Transcriptional regulation of CYP2B1 induction in primary rat hepatocyte cultures: repression by epidermal growth chemical is mediated via a distal enhancer region.** *Mol. Pharmacol.* 65:172-180.

13. [Heder AF](http://ctd.mdibl.org/query.go?type=reference&action=search&partyNmQueryType=equals&partyNm=Heder+AF), Hirsch-Ernst KI, Bauer D, Kahl GF, Desel H (2001) **Induction of cytochrome P450 2B1 by pyrethroids in primary rat hepatocyte cultures.** *Biochem. Pharmacol.* 62:71-79.

14. Eil C, Nisula BC (1990) The binding properties of pyrethroids to human skin fibroblast androgen receptors and to sex hormone binding globulin. [J. Steroid Biochem](javascript:AL_get(this, 'jour', 'J Steroid Biochem.');)*.* 35:409-414.

15. Sakai H, [Iwata H](http://www.ncbi.nlm.nih.gov/sites/entrez?Db=pubmed&Cmd=Search&Term="Iwata H"%5BAuthor%5D&itool=EntrezSystem2.PEntrez.Pubmed.Pubmed_ResultsPanel.Pubmed_DiscoveryPanel.Pubmed_RVAbstractPlus), [Kim EY](http://www.ncbi.nlm.nih.gov/sites/entrez?Db=pubmed&Cmd=Search&Term="Kim EY"%5BAuthor%5D&itool=EntrezSystem2.PEntrez.Pubmed.Pubmed_ResultsPanel.Pubmed_DiscoveryPanel.Pubmed_RVAbstractPlus), [Tsydenova O](http://www.ncbi.nlm.nih.gov/sites/entrez?Db=pubmed&Cmd=Search&Term="Tsydenova O"%5BAuthor%5D&itool=EntrezSystem2.PEntrez.Pubmed.Pubmed_ResultsPanel.Pubmed_DiscoveryPanel.Pubmed_RVAbstractPlus), [Miyazaki N](http://www.ncbi.nlm.nih.gov/sites/entrez?Db=pubmed&Cmd=Search&Term="Miyazaki N"%5BAuthor%5D&itool=EntrezSystem2.PEntrez.Pubmed.Pubmed_ResultsPanel.Pubmed_DiscoveryPanel.Pubmed_RVAbstractPlus) et al. (2006) **Constitutive androstane receptor (CAR) as a potential sensing biomarker of persistent organic pollutants (POPs) in aquatic mammal: molecular characterization expression level and ligand profiling in Baikal seal (Pusa sibirica).** *Toxicol. Sci.* 94:57-70.
