# Supplementary material for: Deciphering Diseases and Biological Targets for Environmental Chemicals using Toxicogenomics Networks
Source: PLoS Comput Biol. 2010 May 20;6(5):e1000788. doi: 10.1371/journal.pcbi.1000788 (PMC2873901; doi:10.1371/journal.pcbi.1000788)
Supplement: Table S1 — Example of molecular target predictions for chemicals. References: 1. Mahgoub AA, El-Medany AH (2001) Evaluation of chronic exposure of the male rat reproductive system to the insecticide methomyl. Pharmacol. Res. 44:73–80. 2. Bernard L, Martinat N, Lécureuil C, Crépieux P, Reiter E, Tilloy-Ellul A, Chevalier S, Guillou F (2007) Dichlorodiphenyltrichloroethane impairs follicle-stimulating hormone receptor-mediated signaling in rat Sertoli cells. Reprod. Toxicol. 23:158–164. 3. Saqib TA, Naqvi SN, Siddiqui PA, Azmi MA (2005) Detection of pesticide residues in muscles liver and fat of 3 species of Labeo found in Kalri and Haleji lakes. J. Environ. Biol. 26:433–438. 4. Flodström S, Hemming H, Warngard L, Ahlborg UG (1990) Promotion of altered hepatic foci development in rat liver cytochrome P450 enzyme induction and inhibition of cell-cell communication by DDT and some structurally related organohalogen pesticides. Carcinogenesis 11:1413–1417. 5. Sakai H, Iwata H, Kim EY, Tsydenova O, Miyazaki N, Petrov EA, Batoev VB, Tanabe S (2006) Constitutive androstane receptor (CAR) as a potential sensing biomarker of persistent organic pollutants (POPs) in aquatic mammal: molecular characterization expression level and ligand profiling in Baikal seal (Pusa sibirica). Toxicol. Sci. 94:57–70 6. Ding X, Staudinger JL (2005) Repression of PXR-mediated induction of hepatic CYP3A gene expression by protein kinase C. Biochem. Pharmacol. 69:867–873. 7. Matsuura I, Saitoh T, Tani E, Wako Y, Iwata H, Toyota N, Ishizuka Y, Namiki M, Hoshino N, Tsuchitani M, Ikeda Y (2005) Evaluation of a two-generation reproduction toxicity study adding endpoints to detect endocrine disrupting activity using lindane. J. Toxicol. Sci. Spec No 135–161. (0.04 MB DOC) [file pcbi.1000788.s007.doc]

| **Chemical** | **Protein candidate** | **Cpscore*** | **Specie** | **Reference** |
| --- | --- | --- | --- | --- |
| Methomyl | LHB | 1.09 | rat | [1] |
| p,p DDT | FSHB | 0.06 | rat | [2] |
|  | GPT | 0.05 | labeo, rat | [3-4] |
|  | NR1I3 | 0.05 | baikal seal | [5] |
| Okadaic acid | CYP3A11 | 1.22 | mice | [6] |
| Lindane | CYP3A11 | 7.41 | rat | [7] |

* Confidence score for each protein established using a neighbor protein procedure.
