# Supplementary material for: Deciphering Diseases and Biological Targets for Environmental Chemicals using Toxicogenomics Networks
Source: PLoS Comput Biol. 2010 May 20;6(5):e1000788. doi: 10.1371/journal.pcbi.1000788 (PMC2873901; doi:10.1371/journal.pcbi.1000788)
Supplement: Table S2 — Illustration of cpscore for approved drugs. (0.09 MB DOC) [file pcbi.1000788.s008.doc]

| **Drug name** | **Proteins known from CTD** | **Proteins known from DrugBank** | **Cpscore*** |
| --- | --- | --- | --- |
| ciprofloxacin | CYP1A2 | CYP1A2 | 4.88 |
|  | [IFNG](http://ctd.mdibl.org/detail.go?type=gene&db=GENE&acc=3458) |  | 4.33 |
|  | [IL4](http://ctd.mdibl.org/detail.go?type=gene&db=GENE&acc=3565) |  | 6.8 |
| citalopram |  | MAOB | 27.71 |
|  | CYP2C19 | CYP2C19 | 2.78 |
|  | CYP2D6 | CYP2D6 | 2.64 |
|  | CYP3A4 |  | 2.35 |
| clozapine | MAOB |  | 34.01 |
|  | HTR1A | HTR1A | 32.93 |
|  | CYP4F2 |  | 30.55 |
|  | INHBE |  | 26.8 |
|  |  | HTR2A | 18.26 |
|  | CYP2D6 | CYP2D6 | 7.33 |
|  | CYP1A2 | CYP1A2 | 5.22 |
| dextromethorphan |  | OPRS1 | 16.48 |
|  | CYPEA5 |  | 13.17 |
|  | CYP2D6 | CYP2D6 | 3.26 |
|  | CYP3A4 |  | 3.22 |
| diclofenac | SLC22A1 |  | 38.57 |
|  | CYP11B1 |  | 22.21 |
|  | CYP2C9 | CYP2C9 | 15.38 |
|  | UGT2B17 |  | 15.15 |
|  | UGT1A1 | UGT1A1 | 12.76 |
|  | CYP2C19 | CYP2C19 | 12.29 |
|  | PTGS1 | PTGS1 | 9.57 |
|  | PTGS2 | PTGS2 | 9.33 |
| fluoxetine | [HTR1A](http://ctd.mdibl.org/detail.go?type=gene&db=GENE&acc=3350) |  | 45.75 |
|  | CYP11B1 |  | 22.21 |
|  | [NR0B2](http://ctd.mdibl.org/detail.go?type=gene&db=GENE&acc=8431) |  | 17.23 |
|  | [NUPR1](http://ctd.mdibl.org/detail.go?type=gene&db=GENE&acc=26471) |  | 12.54 |
|  |  | SLC6A4 | 9.26 |
| haloperidol | CYP2D6 | CYP2D6 | 11.7 |
|  | ABCB1 |  | 8.22 |
| loperamide | CES2 |  | 9.41 |
|  | CES1 |  | 7.12 |
| methadone |  | OPRM1 | 28.7 |
|  | CYP19A1 | CYP19A1 | 14.01 |
|  | CYP3A4 | CYP3A4 | 8.3 |
| sildenafil | PDE5A | PDE5A | 34.8 |
|  |  | PDE2A | 25.4 |
|  | CFTR |  | 3.3 |

* Confidence score for each protein established using our neighbor protein procedure.
